# The effects of contextual diversity on incidental vocabulary learning in the native and a foreign language

**DOI:** 10.1038/s41598-020-70922-1

**Published:** 2020-08-18

**Authors:** Candice Frances, Clara D. Martin, Jon Andoni Duñabeitia

**Affiliations:** 1 Basque Center on Cognition, Brain and Language, BCBL, Donostia, Spain; 2 Department of Social Sciences and Law, UPV/EHU, Donostia, Spain; 3 Centro de Ciencia Cognitiva – C3, Nebrija University, Madrid, Spain; 4 Department of Language and Culture, The Arctic University of Norway, Tromsø, Norway

**Keywords:** Human behaviour, Cognitive neuroscience

## Abstract

Vocabulary learning occurs throughout the lifespan, often implicitly. For foreign language learners, this is particularly challenging as they must acquire a large number of new words with little exposure. In the present study, we explore the effects of contextual diversity—namely, the number of texts a word appears in—on native and foreign language word learning. Participants read several texts that had novel pseudowords replacing high-frequency words. The total number of encounters with the novel words was held constant, but they appeared in 1, 2, 4, or 8 texts. In addition, some participants read the texts in Spanish (their native language) and others in English (their foreign language). We found that increasing contextual diversity improved recall and recognition of the word, as well as the ability to match the word with its meaning while keeping comprehension unimpaired. Using a foreign language only affected performance in the matching task, where participants had to quickly identify the meaning of the word. Results are discussed in the greater context of the word learning and foreign language literature as well as their importance as a teaching tool.

## Introduction

Vocabulary learning is an essential aspect of language that continues throughout the lifespan. To a large extent, the vocabulary we incorporate comes from incidental learning during reading^1,2^ rather than explicit effort. This becomes particularly relevant when learning a new language, where a large amount of vocabulary must be acquired very quickly and partially without supervision. Following this reasoning, several studies have shown that it is possible to learn vocabulary implicitly through reading in our foreign language^3–7^. In addition, we know that people incorporate new lexical forms with as little as one exposure in their native language and as little as two exposures in their foreign language^4^, but that learning improves with exposure to multiple instances of the word^8^.

Implicit word learning in context differs somewhat between one’s native and foreign languages. In particular, reading times for new words in our native language decrease significantly after the first exposure—suggesting some level of incorporation of the lexical item—whereas for the foreign language this happens only after two to four exposures^4^. This might relate to the fact that it is more difficult to extract the meaning of words from context in a foreign language. This is in part because these skills seem to be affected by knowledge of the language and experience in that particular task^9,10^. Although the number of times people encounter a word affects how well they learn and remember it^5,8^, there is not much literature on how spreading these encounters across passages affects learning. One of the ways in which this spread is quantified is through contextual diversity—namely, the number of texts in which a word appears in a database^11,12^. This variable can be used to describe the influence of context beyond the mere number of occurrences or the frequency with which we encounter a given word. Context affects learning of new information^7^, in general, and words^2^, in particular. Contextual diversity specifically has a strong effect on learning^13,14^, as well as on the processing of words, decreasing reaction time in word recognition^11,15^. The effects of contextual diversity have received increasing amounts of attention as they have been found in several domains including spoken word recognition^16^ and serial recall performance with written words^17^.

Word frequency refers to the number of times a word appears in a database, which naturally is highly correlated with the number of texts it appears in^11^, and has been better studied than contextual diversity. Although word frequency has historically been considered a significant predictor of performance in various language-related tasks, such as word learning^8^, lexical access^18^, and serial recall performance^17^, recent studies have questioned this effect. These studies suggest that contextual diversity might be the factor responsible for some of the effects initially attributed to word frequency^12,18,19^, as in some cases it explains more variance than word frequency, rendering the later a non-significant predictor.

Although word frequency and contextual diversity are highly correlated, they show different ERP signatures^19^, suggesting different underlying brain processes, and in some cases, they show opposite behavioral effects. For example, there are cases in which recall is lower for words with high contextual diversity (showing a salience effect) but better for those with high word frequency (showing a benefit of multiple exposures to the word). Furthermore, word frequency—but not contextual diversity—predicts order error, with a higher number of errors for low frequency words, but not for low contextual diversity words^17^. This suggests that the effects of word frequency and contextual diversity may be differentiable in some contexts. In sum, the importance of contextual diversity above and beyond word frequency should not be diminished and its relevance in the context of language learning is still not fully understood.

A recent study by Pagán and Nation^20^ manipulated diversity experimentally by presenting novel words (low frequency unknown words) in repetitions of the same sentence or in different sentences. They found that diversity increased reading times during the learning phase and decreased them during the testing phase. They interpreted this as a processing advantage during testing for words learned in diverse contexts. Although this provided a good first approach to the problem, there were several limitations. Perhaps the more salient one is that repetition, information about the word, and diversity were confounded. By providing the same sentence repeatedly, the reading times for the sentence overall probably decreased, not because of increased comprehension or incorporation of the term but simply due to a repetition effect. In addition, by providing different sentences in the high diversity condition, more information was provided about the meaning of the word. Similarly, they define contextual diversity as repeating (low diversity) or changing (high diversity) sentences when the main and most common definition of this concept is document count^12,21^. In reality, even if a word is repeated in a text, it is not the sentence itself that is repeated, but rather just the word in a new sentence. Another recent study by Rosa, Tapia, and Perea^22^ manipulated contextual diversity by presenting 3rd grade students with different types of texts and found similar improvements in performance on a later test, with higher diversity. In particular, they tested recall, recognition (in two tasks), and picture matching. These tests focused on behavioral differences and showed a consistent benefit in performance for high versus low contextual diversity. All-in-all, these studies set a clear precedent for the importance of assessing the impact of contextual diversity in processing and performance, and the current study is set on these grounds. Here, we specifically tested the relative impact of contextual diversity while keeping frequency constant in foreign language vocabulary learning. To do so, we created several texts incorporating a group of novel words (real words replaced by pseudowords, in this case) to be learned in a nonnative language.

As a first approach to how repetitions should be spread out in our native and foreign languages—namely, the effects of contextual diversity—to improve learning, we had participants read short fictional texts, either in their native or a foreign language. In doing so, we manipulated the contextual diversity of each word, which we defined as the number of texts (or short stories) in which the novel item was encountered. These ranged from very low (eight times in 1 text) to very high (once in each of 8 texts). This allowed us to see not only the effects of contextual diversity on learning, but also whether this affected learning in the native and a foreign language differently.

We expected that distributing exposures in more texts would increase retention overall, as has been seen in other works^4^.Participants were also likely to do better in their native language simply because the ability to derive meaning from context relates to the depth of vocabulary knowledge in that language^10^. This should make it easier for participants to extract the meaning of the pseudowords and incorporate the lexical form. But, if the stimuli are well matched for language difficulty and predictability from context, we may not see overall language effects. Finally, given the increased difficulty in incorporating and making associations between lexical items^23^ in our foreign language, closer repetitions or repetition clusters could help participants extract meaning and incorporate the lexical form in that language^4^. Therefore, spreading might be more beneficial in the native language whereas clustering could be better in the foreign language. On the other hand, if participants are using the same mechanisms in both languages and these are not affected by language ability, we should observe the same contextual diversity effects in both languages.

Our results have practical applications for foreign language vocabulary learning. On the one hand, this study helps determine the importance of spreading practice into several sections (i.e., high contextual diversity) or clustering it (i.e., low or medium contextual diversity). On the other hand, the current research assesses the differences (or lack thereof) between learning in our native or a foreign language.

## Methods

### Participants

Using GPower^24^, we ran an a priori power analysis based on prior studies^22,25^ and a medium size effect (*η*_*p*_^2^ = 0.06) to establish sample size. We determined a minimum requirement of 80 participants to have 80% power.

Participants were 88 native Spanish speakers (44 in each language group, 25 males, M_age_ = 23.78, SD_age_ = 4.28). These were recruited through the internal database at the Basque Center on Cognition, Brain and Language (BCBL) and randomly assigned to either the native language (NL) or foreign language (FL) condition. All participants completed a test of receptive English and Spanish vocabularies (LexTALE^26^ and LexTALE-Esp^27^). We only included participants with minimum score of 60% in English (80% for Spanish), which is equivalent to a minimum of a B2 level (C1 for Spanish) according to the Common European Framework of reference for languages^26^. Participants also completed a test of productive vocabulary (BEST picture naming task^28^) and had a minimum score of 40 out of 65 for English (61 out of 65 for Spanish). Participants were asked to rate their English and Spanish levels overall on a 1-to-10 scale as well as their reading skills in that language. They also reported their estimated age of acquisition of each language and had a minimum age of 3 years for English and a maximum of 3 years for Spanish. Participants reported their daily exposure to each language, their educational level (highest level of schooling achieved, in all cases at least high school), and student status. And, finally, we collected measures of verbal, nonverbal, and compound IQ^29^. Participants were matched between groups on all of the above-mentioned variables. For a summary of these variables, see Table 1. All participants gave written informed consent and were compensated 8€ for their time. The study and protocols were approved by the ethics committee at the BCBL (approval number 11049) and were conducted in accordance with the Declaration of Helsinki.

**Table 1:** Means, standard deviations, and statistics for participants.

Variable	Overall	English	Spanish	Statistic	Bayes Factor
Age	23.78 (4.28)	23.91 (4.70)	23.66 (3.87)	*t*(86) = 0.27, *p* = 0.79	BF_01_ = 4.34, Error % = 0.03
English LexTALE	71.28 (8.13)	70.43 (8.14)	72.151 (8.13)	*t*(86) = -0.90, *p* = 0.37	BF_01_ = 3.14, Error % = 0.03
Spanish LexTALE	95.20 (4.05)	95.61 (4.02)	94.79 (4.08)	*t*(86) = 0.94, *p* = 0.35	BF_01_ = 3.04, Error % = 0.03
English BEST	51.10 (6.87)	50.82 (6.574)	51.39 (7.21)	*t*(86) = -0.39, *p* = 0.70	BF_01_ = 4.20, Error % = 0.03
Spanish BEST	64.72 (0.68)	64.66 (0.81)	64.77 (0.52)	*t*(86) = -0.79, *p* = 0.43	BF_01_ = 3.42, Error % = 0.03
English level (overall)	6.99 (1.89)	7.00 (2.04)	6.98 (1.76)	*t*(81) = 0.06, *p* = 0.96	BF_01_ = 4.37, Error % = 0.03
English level (reading)	7.48 (2.18)	7.39 (2.20)	7.57 (2.19)	*t*(81) = -0.38, *p* = 0.71	BF_01_ = 4.11, Error % = 0.03
Spanish level (overall)	9.21 (1.67)	9.27 (1.62)	9.14 (1.73)	*t*(86) = 0.38, *p* = 0.70	BF_01_ = 4.21, Error % = 0.03
Spanish level (reading)	8.98 (2.38)	9.11 (2.22)	8.84 (2.54)	*t*(86) = 0.54, *p* = 0.60	BF_01_ = 3.95, Error % = 0.03
AOA English	5.81 (2.08)	5.82 (1.81)	5.80 (2.35)	*t*(86) = 0.05, *p* = 0.96	BF_01_ = 4.48, Error % = 0.03
AOA Spanish	0.17 (0.65)	0.14 (0.63)	0.21 (0.67)	*t*(86) = -0.49, *p* = 0.62	BF_01_ = 4.03, Error % = 0.03
Daily usage English	14.82 (9.67)	13.90 (8.02)	15.71 (11.08)	*t*(81) = -0.85, *p* = 0.40	BF_01_ = 3.19, Error % = 0.03
Daily usage Spanish	59.32 (16.39)	58.86 (16.17)	59.77 (16.77)	*t*(86) = -0.26, *p* = 0.80	BF_01_ = 4.36, Error % = 0.03
Verbal IQ	101.60 (22.50)	101.21 (24.25)	102.00 (20.88)	*t*(86) = -0.17, *p* = 0.87	BF_01_ = 4.43, Error % = 0.03
Non-verbal IQ	108.60 (18.58)	108.55 (18.63)	108.66 (18.75)	*t*(86) = -0.03, *p* = 0.98	BF_01_ = 4.48, Error % = 0.03
Compound IQ	103.50 (21.62)	102.61 (23.98)	104.39 (19.21)	*t*(86) = -0.38, *p* = 0.70	BF_01_ = 4.21, Error % = 0.03
Gender	88 (63)	44 (32)	44 (31)	*Χ*^2^(1) = 0.06, *p* = 0.81	BF_01_ = 4.13
Handedness	88 (13)	88 (8)	88 (5)	*Χ*^2^(1) = 0.74, *p* = 0.39	BF_01_ = 3.75
Educational level	88	88	88	*Χ*^2^(3) = 0.40, *p* = 0.94	BF_01_ = 28.47
Student status	88 (67)	44 (32)	44 (35)	*Χ*^2^(1) = 0.56, *p* = 0.45	BF_01_ = 3.12

Values in the Overall, English, and Spanish columns represent means and standard deviations (in parentheses). For gender, values represent count and number of females (in parentheses). For handedness, values represent count and number of left-handed people (in parentheses). For student status, values represent count and number of participants currently enrolled at a university (in parentheses). For educational level, only total count is presented because this was not a dichotomous variable (levels: high school, professional training, university, and graduate school).

### Stimuli

Stimuli consisted of 100-word stories using 8 high frequency words (hereafter, keywords) that were one of the most representative exemplars of their category group^30^: fruit (apple—*manzana*), vehicle (car—*coche*), furniture (table—*mesa*), animal (dog—*perro*), dwelling (house—*casa*), reading material (book—*libro*), beverage (water—*agua*), and toy (ball—*balón*). These high frequency words would later be replaced by pseudowords.

Our choice of stimuli was motivated by several reasons. We needed stimuli that could (1) be easily understood and deduced from the sentences they were contained in, (2) apply to a variety of scenarios—as each one would appear in a set of 15 different stories, and (3) be easily identifiable from a picture. Given the current design, we needed that each sentence provided by itself enough information for participants to fully comprehend the critical word without the need of a greater context. Besides, it should be kept in mind that the selected items should fit the native and foreign language conditions, and choosing medium or low frequency words would hardly represent a good option, since they would presumably be unknown to most or some participants in the foreign language, making the two language conditions unbalanced. Hence, by choosing very high frequency words, we could make sure that they were known in both languages, easily deduced from the sentence context, applicable to a variety of scenarios, and easily depicted by standardized images for the recognition tests.

**Figure 1:**
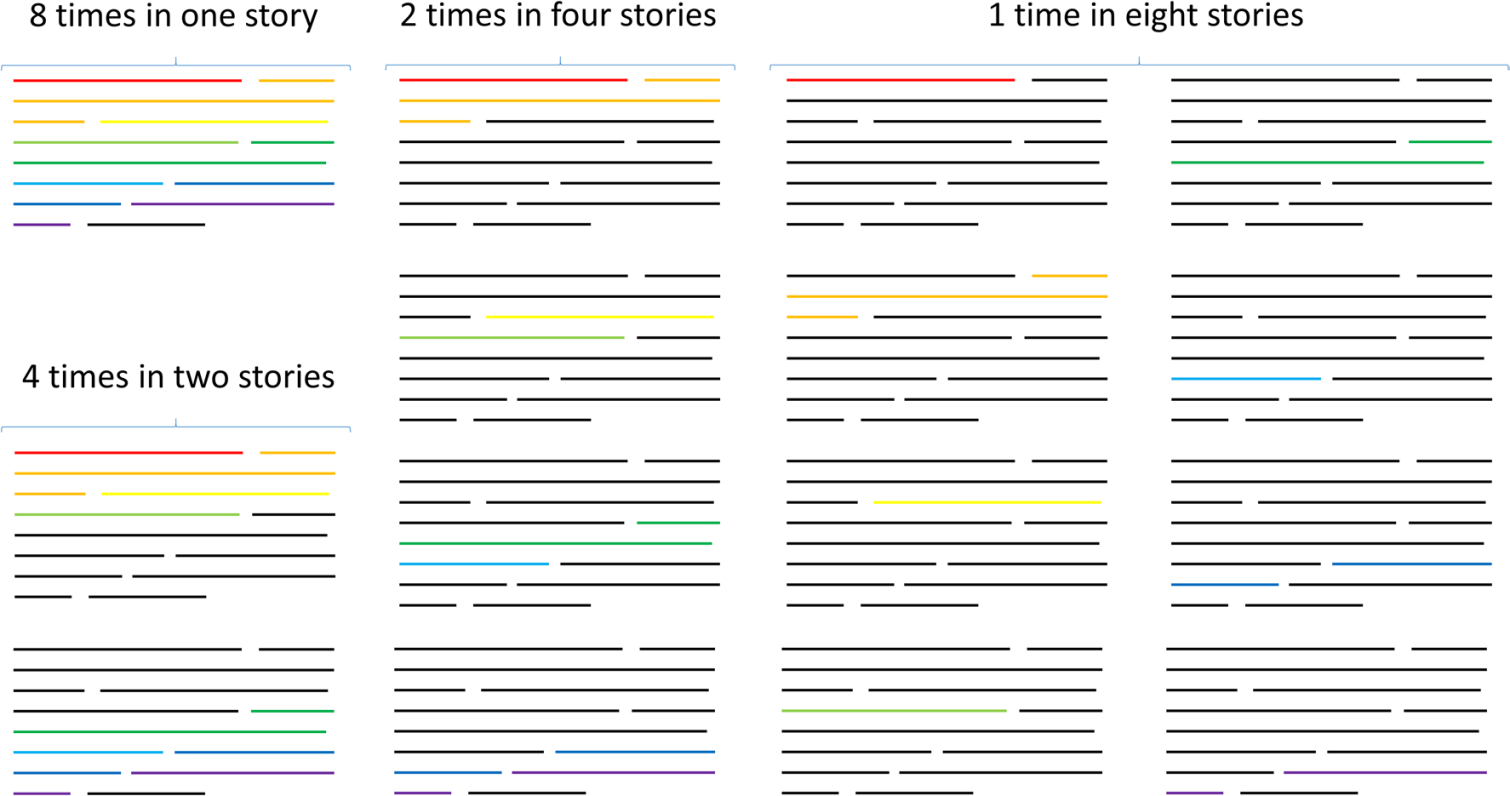
Schematic representation of how the stimulus stories were created. The color lines represent different sentences with the keyword and the black lines represent filler sentences (i.e., sentences that did not contain the keyword). Each text ended with a filler sentence and had a total of nine sentences each. First, the text with the term eight times was created. Then, this text was subdivided into two to create the two texts with the term four times. Each of these texts contained the term in four consecutive sentences that were exactly the same, in the same order and placement in the text as in the original story. The stories were then completed with filler sentences to reach nine sentences (respecting the original placement of the sentences containing the keywords). The original story was then subdivided in a similar fashion to create the four stories with the keyword twice and the eight stories with the keyword once.

Each story contained the keyword eight times (1 story), four times (2 stories), twice (4 stories), or just once (8 stories)—see Supplementary Table S1. The stories were created so that the story with the keyword eight times contained it in eight consecutive sentences and ended with a filler sentence (meaning a sentence without the keyword—see Supplementary Table S2 for a list of key sentences). Then, the sentences with the keyword were subdivided and filler sentences were added before and/or after in order to create the remaining texts (see Fig. 1 for a schematic representation of how the stimuli were created and Supplementary Table S3 for a worked out example). Although the other sentences were fillers, they did compose a cohesive paragraph. This way, the sentences containing the keyword were the same between conditions. These stories were then translated to create an English and a Spanish version, matched for word count. For each of the sentences containing the keyword, we carried out a norming study to assure that the predictability for the keywords was high (for Spanish [N = 9]: M = 81%, SD = 19%; for English L1 [N = 15]: M = 78%, SD = 22%; for English L2 [N = 9]: M = 64%, SD = 23%). Then, we replaced the keyword with a pseudoword of the form CVCVC, VCVCV, or VCVC, matched for bigram frequency (calculated using B-Pal^31^ for Spanish and N-Watch^32^ for English: bigram frequency mean token, *t*(7) = 1.56, *p* = 0.16; bigram frequency mean type, *t*(7) = 0.96, *p* = 0.37) and plausibility (rated from 1 to 5 by 14 native Spanish speakers, the average rating by item was not significantly different, *t*(7) = 1.23, *p* = 0.26) between languages. The pseudoword replacing the keyword in each story was the new target word to be learned during the task.

### Procedure

Each participant was assigned either to the native language (Spanish; NL) or foreign language (English; FL) condition. Participants in each language condition were given all instructions in that language, both orally and on the screen, so as to avoid language switching effects. To assure comprehension, participants were given the instructions both orally and in written form. For the learning task, participants were given a practice trial and for the testing phase they were shown examples. All of the tasks were carried out using OpenSesame^33^.

**Figure 2:**
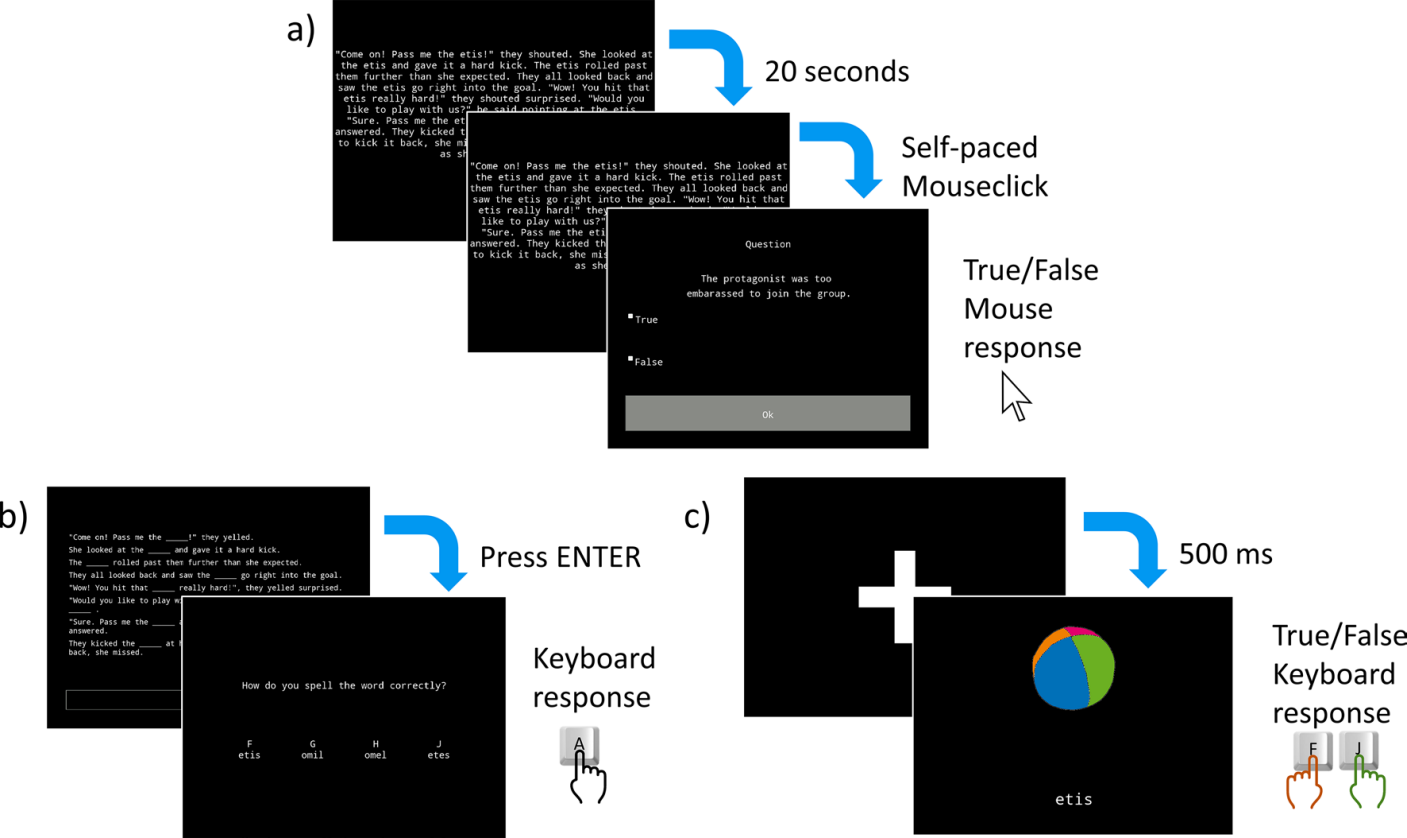
Schematic representation of the procedure. (**a**) Learning stage in which participants read each of the 30 texts. (**b**) Recall (fill in the blank) task which led to the recognition task, which required an untimed keyboard response. (**c**) Matching task in which participants were shown image-word pairs and were asked to determine whether they matched in meaning or not.

During the learning phase, participants were presented with texts and asked to read for comprehension. These texts contained eight novel pseudowords—two per diversity condition embedded in 30 stories (two with a pseudoword repeated eight times, four with a pseudoword repeated four times each, eight with a pseudoword repeated twice each, and 16 with a pseudoword only once per story). Participants were warned that there would be strange words in the texts and were advised to focus on reading for comprehension rather than focusing on those words. They then read one example text (before the 30 experimental texts) which contained a pseudoword they were not tested on, and were asked to answer two practice comprehension questions (see Fig. 2a and Supplementary Figure S4). The full list of texts (regardless of condition) was randomized. In order to avoid primacy effects on the conditions with the fewest number of texts (i.e., the condition with only one text with the pseudoword eight times and the one where there were two texts with the pseudoword four times) one of the eight texts from the highest diversity condition was put in first position. Similarly, to avoid recency effects we took one text from the other pseudoword in the highest diversity condition and placed it last as well as adding a distractor task before the testing portion. The other 28 texts were presented to each participant in a fully randomized order. While reading each story they were not allowed to continue until at least 15 s had passed. Once those 15 s had passed, an arrow appeared that the participant could press at any time (self-paced) to continue. After reading each text, participants answered one true–false comprehension question (not containing the novel pseudowords) to test both for attention and comprehension (comprehension check).

Once they had read all of the texts, participants completed a distractor task, which was the forwards and backwards Corsi Task^34^, with a 10-min timer to assure that they all had equal-length breaks. After the distractor task, participants entered the testing phase, which consisted of a recall task, a recognition task, and a matching task (similar to those used by Rosa et al.^22^). For the recall task, they were presented with the eight sentences they had seen before in which the pseudoword appeared, except that blanks (lines) were placed where the pseudowords had been. Each sentence was presented on a separate line and in order, with the entirety of the text aligned left, but occupying most of the screen. Underneath, there was a rectangle in which participants were asked to type in the correct pseudoword that completed all of the sentences (see Fig. 2b and Supplementary Figure S5). Immediately after each fill-in-the-blank, they did the recognition task, which consisted of a multiple-choice question corresponding to the same pseudoword. They were presented with four options (the correct pseudoword, a competitor pseudoword, and two versions of these with middle consonants transposed) (see Fig. 2b and Supplementary Figure S5). Both of these tasks were self-paced and the order of words was fully randomized, while keeping the order of tasks constant (first recall and then recognition). After completing the recall and recognition for the first word, they proceeded similarly for the remaining seven pseudowords.

After participants had completed the recall and recognition tasks, they were asked to complete the matching task. They were presented with a drawing of a real object (centered horizontally but with its center on the one-third mark vertically), and a pseudoword (centered horizontally but with its center on the bottom one third, vertically) and were asked to say whether they matched (i.e., if the letter string meant the object) or not (see Fig. 2c and Supplementary Figure S6). The drawings were extracted from the MultiPic database^35^ and depicted the high frequency words (with the exception of “water” for which we used the image for “faucet”). These images represented either the real object that was replaced by the pseudoword, a category competitor, a related word, or an unrelated image (i.e., the category competitor for a different pseudoword)—see Supplementary Table S1 for the full list and Supplementary Figure S6 for how it looked. They had 2500 ms to respond with the F and J keys on the keyboard for not-matching and matching decisions, respectively. Stimuli were presented in random order (see Fig. 2 for a schematic representation of the procedure).

## Results

In all cases, alpha was set at 0.05. All t-tests reported are two-tailed. The number of participants (n) in all cases was 88. In all cases, the data was verified not to violate assumptions of normality. All analyses were run using JASP^36^.

For the recall task, we also utilized the ALINE distance measure. ALINE distance is a measure of string alignment, which aligns phoneme strings, quantifying and standardizing the number of operations (insertions/deletions, substitutions, and expansions/compressions) necessary for going from one string to the other taking into account the features of the phonemes it compares^37^. This measures similarity between strings on a scale from 0 to 1, with lower scores showing increasing difference and one being exactly the same string. We calculated the ALINE similarity score between each item produced by the participant and the correct answer^38^ using the alineR package^39^ for R^40,41^. For these calculations, we removed any item that was shorter than 3 characters long and any items in which the participant produced the real word as opposed to the pseudoword, as these were not considered real attempts.

### Comprehension check and reading times

The average accuracy score was 88% (SD = 8.4%). We carried out a two-way mixed ANOVA with Diversity (1, 2, 4, and 8 texts) and Language (foreign and native) on the performance on the comprehension test. There were no main effects of Language [*F*_1_(1,86) = 0.77, *p* = 0.38, *η*_*p*_^2^ = 0.01, BF_01_ = 4.65 , error% = 0.69; *F*_2_(1,7) = 1.82, *p* = 0.22, *η*_*p*_^2^ = 0.21, BF_01_ = 3.88, error% = 1.21] or Diversity [*F*_1_(3,258) = 0.46, *p* = 0.71, *η*_*p*_^2^ = 0.01, BF_01_ = 43.88, error% = 0.36; *F*_2_(3,21) = 0.07, *p* = 0.98, *η*_*p*_^2^ = 0.01, BF_01_ = 10.10, error% = 0.72] and no interaction [*F*_1_(3,258) = 1.75, *p* = 0.16, *η*_*p*_^2^ = 0.02, BF_01_ = 3.88, error% = 1.43; *F*_2_(3,21) = 0.24, *p* = 0.87, *η*_*p*_^2^ = 0.03, BF_01_ = 5.38, error% = 3.73] (see Table 2).

**Table 2:** Means, standard errors of the mean (numbers in parentheses), and 95% confidence intervals (values between brackets) by language for each of the tasks.

	8 texts	4 texts	2 texts	1 text
**Comprehension**				
Spanish	0.88 (0.02) [0.85; 0.92]	0.93 (0.02) [0.88; 0.98]	0.88 (0.02) [0.84; 0.92]	0.89 (0.04) [0.81; 0.96]
English	0.86 (0.01) [0.83; 0.89]	0.85 (0.02) [0.82; 0.89]	0.88 (0.02) [0.83; 0.93]	0.91 (0.03) [0.84; 0.98]
**Recall (accuracy)**				
Spanish	0.28 (0.05) [0.18; 0.39]	0.16 (0.05) [0.07; 0.25]	0.13 (0.03) [0.06; 0.19]	0.03 (0.02) [0.00; 0.07]
English	0.30 (0.05) [0.19; 0.40]	0.22 (0.05) [0.12; 0.32]	0.17 (0.05) [0.07; 0.27]	0.03 (0.02) [0.00; 0.07]
**Recall (aline)**				
Spanish	0.62 (0.05) [0.53; 0.71]	0.47 (0.05) [0.37; 0.57]	0.49 (0.04) [0.41; 0.58]	0.38 (0.04) [0.30; 0.46]
English	0.61 (0.05) [0.51; 0.72]	0.54 (0.05) [0.43; 0.65]	0.51 (0.05) [0.40; 0.61]	0.42 (0.04) [0.34; 0.50]
**Recognition**				
Spanish	0.82 (0.04) [0.74; 0.90]	0.75 (0.04) [0.66; 0.84]	0.82 (0.04) [0.73; 0.90]	0.63 (0.05) [0.53; 0.72]
English	0.89 (0.04) [0.81; 0.96]	0.74 (0.05) [0.63; 0.84]	0.80 (0.05) [0.70; 0.89]	0.59 (0.06) [0.48; 0.70]
**Matching (accuracy)**				
Spanish	0.77 (0.03) [0.71; 0.83]	0.71 (0.03) [0.65; 0.77]	0.75 (0.03) [0.69; 0.81]	0.71 (0.02) [0.66; 0.75]
English	0.70 (0.03) [0.64; 0.76]	0.66 (0.03) [0.60; 0.72]	0.64 (0.03) [0.58; 0.71]	0.62 (0.03) [0.56; 0.68]
**Matching (response time)**				
Spanish	1244.83 (40.87) [1,163.08; 1326.57]	1275.74 (35.13) [1205.47; 1346.00]	1289.63 (34.40) [1220.82; 1358.44]	1329.57 (35.39) [1258.78; 1400.35]
English	1285.50 (29.37) [1226.77; 1344.24]	1367.09 (38.48) [1290.12; 1444.06]	1366.06 (38.13) [1289.81; 1442.32]	1378.91 (41.19) [1296.53; 1461.29]

Ranges of possible values for Comprehension, Recall, Recognition, and Matching accuracy are all 0 to 1. For Matching response time, ranges were 200 to 2500 ms.

The average time people took to read each paragraph was 38.05 s (SD = 9.84 s). We carried out a two-way mixed ANOVA with Diversity (1, 2, 4, and 8 texts) and Language (foreign and native) on reading times during learning. There was a main effect of Language [*F*_1_(1,86) = 28.74, *p* < 0.001, *η*_*p*_^2^ = 0.25, BF_01_ = 6.32 × 10^–5^ , error% = 1.69 × 10^–7^; *F*_2_(1,7) = 275.13, *p* < 0.001, *η*_*p*_^2^ = 0.98, BF_01_ = 1.33 × 10^–14^, error% = 2.95], with participants taking longer to read in the foreign language (M = 42.91 s; SD = 10.10 s) than in the native one (M = 33.19 s; SD = 6.75 s). There was no main effect of Diversity [*F*_1_(3,258) = 0.98, *p* = 0.40, *η*_*p*_^2^ = 0.01, BF_01_ = 23.23, error% = 3.03; *F*_2_(3,21) = 0.13, *p* = 0.94, *η*_*p*_^2^ = 0.02, BF_01_ = 11.148, error% = 0.57] and no interaction [*F*_1_(3,258) = 0.32, *p* = 0.81, *η*_*p*_^2^ = 0.004, BF_01_ = 24.51, error% = 2.29; *F*_2_(3,21) = 0.24, *p* = 0.87, *η*_*p*_^2^ = 0.03, BF_01_ = 6.08, error% = 2.60]. For the foreign language, reading times correlated negatively with accuracy in the recall task (*r*(43) = − 0.43, *p* = 0.004) and the recognition task (A’: *r*(43) = − 0.33, *p* = 0.03), as well as positively with response time in the matching task (*r*(43) = 0.64, *p* < 0.001) and the recognition task (*r*(43) = 0.50, *p* < 0.001), but not with ALINE distance in the recall task (*r*(41) = − 0.28, *p* = 0.07) nor with A’ in the matching task (*r*(43) = − 0.23, *p* = 0.13). For the native language, reading times only correlated with response time in the recognition task (*r*(43) = 0.32, *p* = 0.04) and marginally with response time in the matching task (*r*(43) = 0.27, *p* = 0.07), but not with any of the other measures (*p’*s > 0.4).

### Recall task

The recall data (both accuracy and ALINE distance) was not normally distributed. To correct for the non-normality of the data, we carried out non-parametric tests—see Supplementary Table S4, but the results were the same as the frequentists and Bayesian tests. For homogeneity of analysis and for simplicity, here, we report the frequentist analyses.

**Figure 3:**
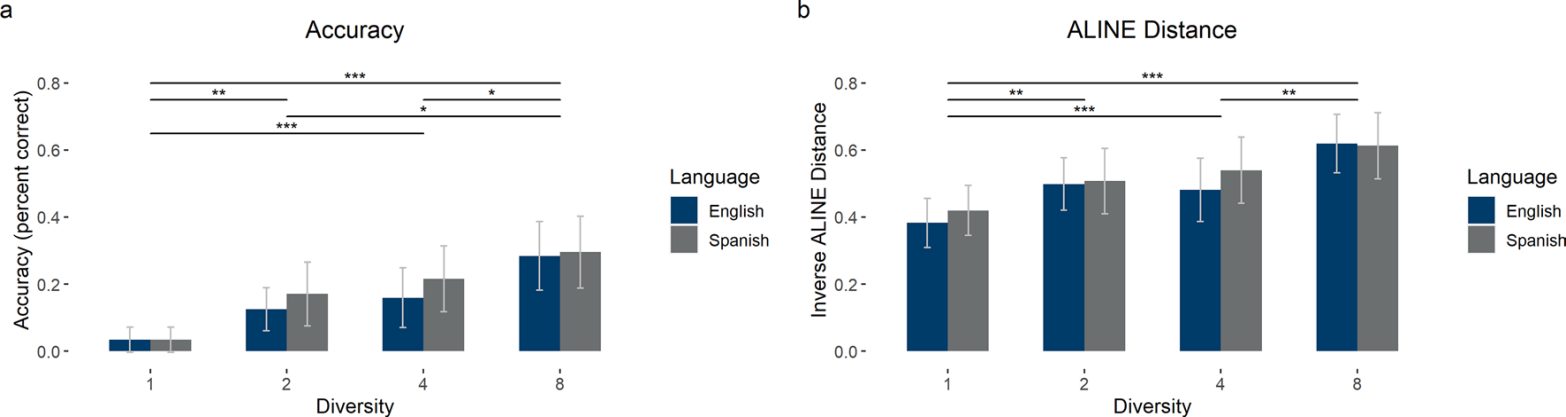
Recall task results. (**a**) Accuracy in the recall task by language and contextual diversity condition. (**b**) Average inverse Aline distance in the recall task by language and contextual diversity condition. Error bars represent 95% confidence intervals. Horizontal lines show significant pairwise comparisons for the main effect of diversity. *p < .05; **p < .01; ***p < .001.

For this part of the analysis, we only considered pseudowords that were correctly recalled—pseudowords for which the produced string matched perfectly with the target. On average, recall was fairly low (M = 17.8%, SD = 16.0%). We carried out a two-way mixed ANOVA with Diversity and Language on accuracy—namely, percent correct—in the recall task. There was no main effect of Language (M_Eng_ = 15.1%, SD_Eng_ = 14.7%; M_Spa_ = 17.9%, SD_Spa_ = 17.3%) [*F*_1_(1,86) = 0.69, *p* = 0.41, *η*_*p*_^2^ = 0.01, BF_01_ = 5.07, error% = 1.43; *F*_2_(1,7) = 2.00, *p* = 0.20, *η*_*p*_^2^ = 0.22, BF_01_ = 2.92, error% = 1.33], but there was a main effect of Diversity [*F*_1_(3,258) = 13.71, *p* < 0.001, *η*_*p*_^2^ = 0.14, BF_01_ = 6.72 × 10^–7^, error% = 13.16; *F*_2_(3,21) = 10.67, *p* < 0.001, *η*_*p*_^2^ = 0.60, BF_01_ = 1.52 × 10^–5^, error% = 0.46], such that items presented with greater diversity were recalled better (see Table 2). There was no interaction [*F*_1_(3,258) = 0.22, *p* = 0.88, *η*_*p*_^2^ < 0.01, BF_01_ = 25.53, error% = 1.95; *F*_2_(3,21) = 0.32, *p* = 0.81, *η*_*p*_^2^ = 0.04, BF_01_ = 5.43, error% = 5.81]. See Fig. 3.

Given the difficulty of the task, we also analyzed partial recall—pseudowords that were partially, but not completely correct. In order to quantify this partial recall, we used the ALINE similarity score (one minus the ALINE distance). Using those data, we carried out a two-way mixed ANOVA with Diversity and Language on Aline similarity in the recall task. The average Aline similarity score was 0.51 (SD = 0.23). There was no main effect of Language [*F*_1_(1,86) = 0.29, *p* = 0.59, *η*_*p*_^2^ < 0.01, BF_01_ = 3.82, error% = 0.61; *F*_2_(1,7) = 1.29, *p* = 0.29, *η*_*p*_^2^ = 0.16, BF_01_ = 2.79, error% = 1.40], but there was a main effect of Diversity [*F*_1_(3,258) = 13.65, *p* < 0.001, *η*_*p*_^2^ = 0.15, BF_01_ = 2.17 × 10^–6^, error% = 1.13; *F*_2_(3,21) = 9.33, *p* < 0.001, *η*_*p*_^2^ = 0.57, BF_01_ = 3.04 × 10^–4^, error% = 0.58], such that items presented with greater diversity elicited strings closer to the correct pseudoword (see Table 2 and Fig. 3). There was no interaction [*F*_1_(3,258) = 0.45, *p* = 0.72, *η*_*p*_^2^ = 0.01, BF_01_ = 18.43, error% = 1.55; *F*_2_(3,21) = 0.34, *p* = 0.80, *η*_*p*_^2^ = 0.05, BF_01_ = 5.20, error% = 1.86].

### Recognition task

The recognition accuracy data was not normally distributed. To correct for the non-normality of the data, we carried out non-parametric tests—see Supplementary Table S4—, but the results were the same as the frequentists and Bayesian tests. For homogeneity of analysis and for simplicity, here, we report the frequentist analyses.

**Figure 4:**
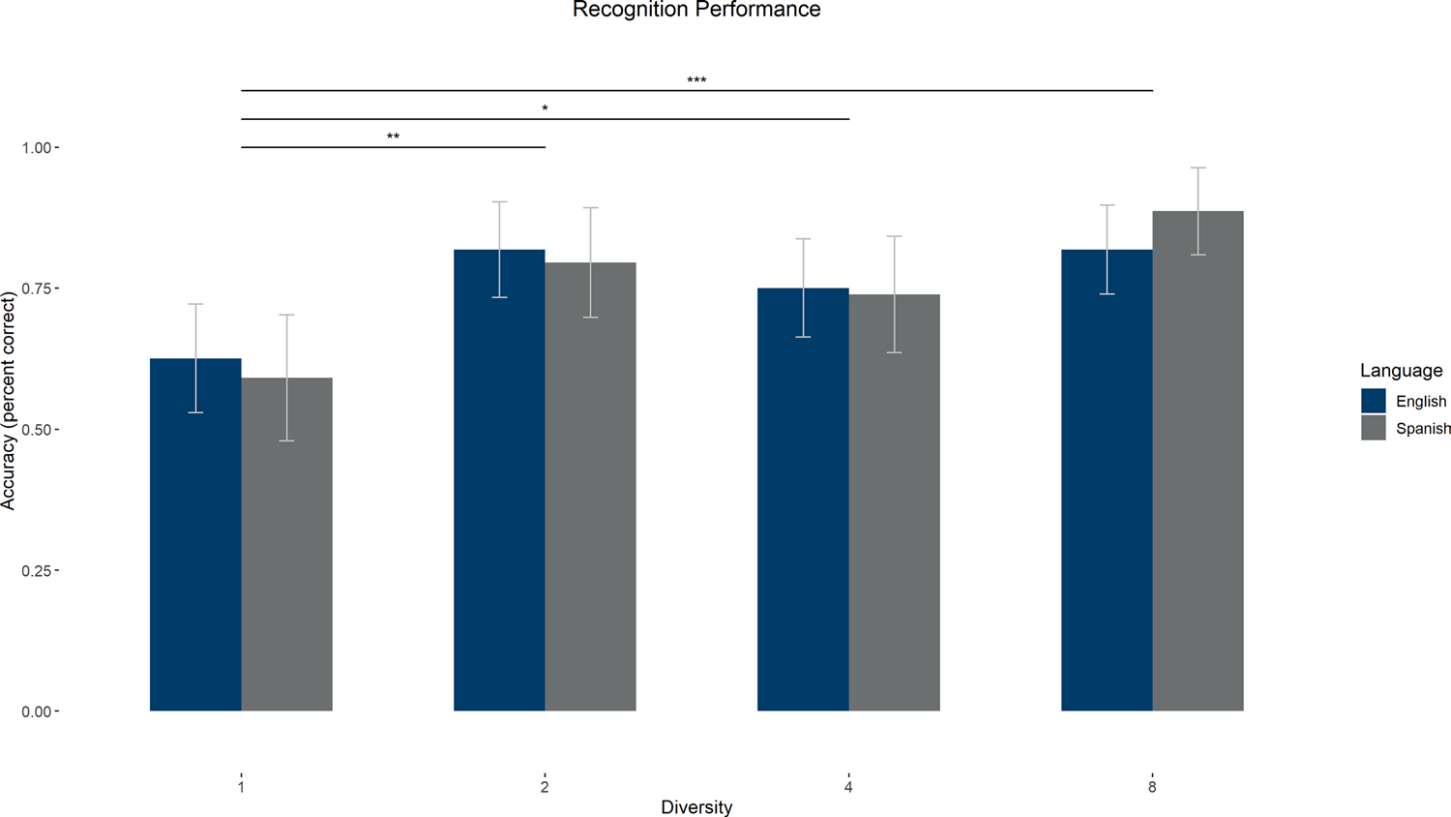
Recognition task results. Accuracy in the recognition task by language and contextual diversity condition. Error bars represent 95% confidence intervals. Brackets show significant pairwise comparisons for the main effect of diversity. *p < .05; **p < .01; ***p < .001.

On the recognition task, the average correct recognition score was 75.28% (SD = 16.08%), with chance being 25%. We carried out a two-way mixed ANOVA with Diversity and Language on accuracy on the recognition task. There was a main effect of Diversity [*F*_1_(3,258) = 10.30, *p* < 0.001, *η*_*p*_^2^ = 0.11, BF_01_ = 5.77 × 10^–6^, error% = 0.57; *F*_2_(3,21) = 9.54, *p* < 0.001, *η*_*p*_^2^ = 0.58, BF_01_ = 1.44 × 10^–4^, error% = 0.73], but no main effect of Language [*F*_1_(1,86) = 9.14 × 10^–31^, *p* = 1, *η*_*p*_^2^ < 0.01, BF_01_ = 6.80, error% = 1.61; *F*_2_(1,7) = 0.003, *p* = 0.96, *η*_*p*_^2^ < 0.001, BF_01_ = 4.01, error% = 1.15] and no interaction [*F*_1_(3,258) = 0.49, *p* = 0.69, *η*_*p*_^2^ = 0.01, BF_01_ = 18.86, error% = 1.97; *F*_2_(3,21) = 0.76, *p* = 0.53, *η*_*p*_^2^ = 0.10, BF_01_ = 4.16, error% = 5.04]. The main effects showed that items presented with greater diversity were recognized better (see Table 2 and Fig. 4).

### Matching task

**Figure 5:**
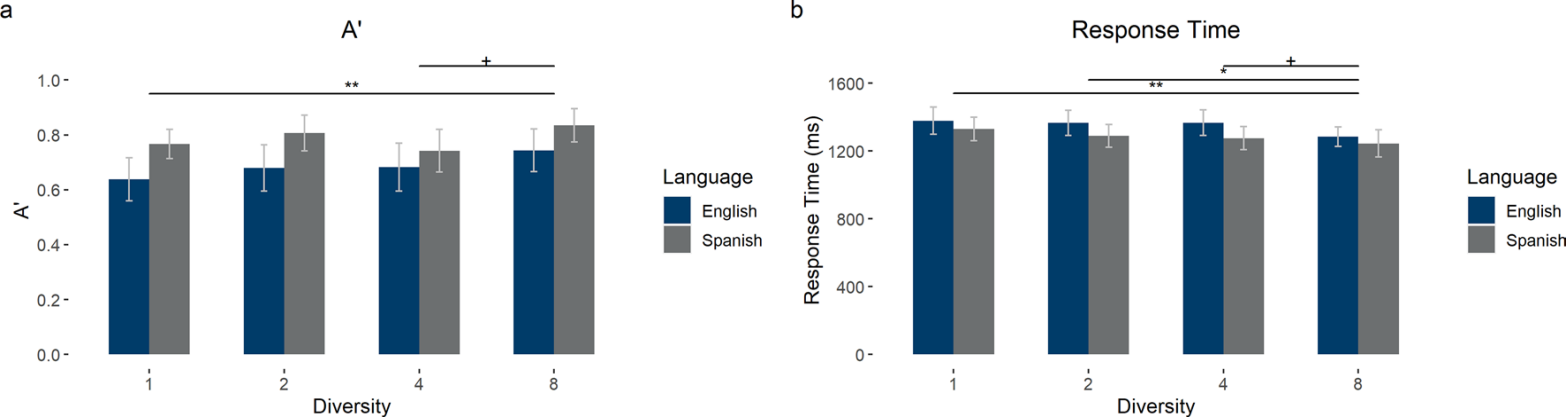
Matching task results. (**a**) A’ in the matching task by language and contextual diversity condition. (**b**) Response times (RT) in the matching task by language and contextual diversity condition. Error bars represent 95% confidence intervals. Horizontal lines show significant pairwise comparisons for the main effect of diversity. ^+^*p* < .1; *p < .05; **p < .01; ***p < .001.

Overall, accuracy in the matching task was 69.97% (SD = 14.96), with chance being 50%. We calculated A’—a sensitivity index that takes into consideration hits and false alarms—using the Psycho package in R^42^. We carried out a two-way mixed ANOVA with Diversity and Language on A’ on the matching task. There were main effects of Language [*F*_1_(1,86) = 6.75, *p* = 0.01, *η*_*p*_^2^ = 0.07, BF_01_ = 0.26, error% = 0.42; *F*_2_(1,7) = 26.46, *p* < 0.001, *η*_*p*_^2^ = 0.79, BF_01_ = 0.005, error% = 1.01] and Diversity [*F*_1_(3,258) = 3.51, *p* = 0.02, *η*_*p*_^2^ = 0.04, BF_01_ = 0.88, error% = 0.25; *F*_2_(3,21) = 3.04, *p* = 0.05, *η*_*p*_^2^ = 0.30, BF_01_ = 0.91, error% = 0.51], but no interaction [*F*_1_(3,258) = 0.63, *p* = 0.60, *η*_*p*_^2^ = 0.01, BF_01_ = 16.16, error% = 0.96; *F*_2_(3,21) = 0.41, *p* = 0.75, *η*_*p*_^2^ = 0.06, BF_01_ = 4.79, error% = 2.01]. The main effects showed that participants in the native language condition had better signal detection—i.e., a combination of more hits and fewer false alarms—than those in the foreign language condition and that items presented with greater diversity were matched with greater discrimination ability (see Table 2 and Fig. 5).

We also carried out a two-way mixed ANOVA with Diversity and Language on response time on the matching task. There was a main effect of Diversity [*F*_1_(3,258) = 5.11, *p* = 0.002, *η*_*p*_^2^ = 0.06, BF_01_ = 0.10, error% = 2.86; *F*_2_(3,21) = 4.11, *p* = 0.02, *η*_*p*_^2^ = 0.37, BF_01_ = 0.38, error% = 0.69], but no main effect of Language in the by participant analysis (although it does show up in the by item analysis, with response times in the foreign language being longer) [*F*_1_(1,86) = 2.21, *p* = 0.14, *η*_*p*_^2^ = 0.03, BF_01_ = 1.47, error% = 0.35; *F*_2_(1,7) = 34.49, *p* < 0.001, *η*_*p*_^2^ = 0.83, BF_01_ = 0.04, error% = 1.07] and no interaction [*F*_1_(3,258) = 0.50, *p* = 0.68, *η*_*p*_^2^ = 0.01, BF_01_ = 19.15, error% = 1.73; *F*_2_(3,21) = 0.14, *p* = 0.93, *η*_*p*_^2^ = 0.02, BF_01_ = 5.58, error% = 2.51]. The main effect of Diversity showed that items presented with greater diversity were responded to faster (see Table 2 and Fig. 5).

## Discussion

The purpose of this study was to expand our knowledge of incidental learning. In particular, we tested whether the distribution of encounters with a new word in one or several texts affected learning, paying particular attention to the comparison between native and foreign language vocabulary learning. With this purpose in mind, we had participants read short stories with pseudowords replacing high frequency words. Those pseudowords could appear in fewer or more texts, being associated with lower or higher diversity, respectively. In addition, participants were performing the task either in their native (Spanish) or a foreign (English) language. Participants had to answer a question after every text and showed no effect of language or diversity in this comprehension check. We then had participants recall and recognize those pseudowords as well as match them with the objects they represented.

Overall, we found a main effect of contextual diversity in all tasks, with participants performing better—faster and/or more accurately—with pseudowords that they had seen in more contexts. This means that, in the full absence of comprehension problems, diversity only had a positive impact, making the pseudowords easier to recall, recognize, and match with their meaning. Our results are in line with prior studies that show effects of diversity above and beyond those of frequency^4,20,22^. This suggests that simply manipulating contextual diversity might be enough to improve performance without increasing frequency of exposure. It should be noted that in addition to increasing contextual diversity, our manipulation increased spacing between encounters, which might have also boosted the effects and had a positive effect on retention. Nevertheless, spacing literature refers to separate sessions, often carried out on different days. As an example, Sobel, Cepeda, and Kapler ^43^ used a 10 min task with just one minute between sessions in the massed condition, and with one week between sessions in the spaced condition. Whereas all of our conditions could be considered massed according to this view, it should be noted that our manipulations do not fit strict definitions of massed and spaced exposure, since words were never repeated consecutively—at most they were in consecutive sentences—and they were never spaced in separate sessions—each participant had only one session. Word meanings are created through the summation of experiences with a word and the words it co-occurs with ^44,45^. Hence, while spacing and diversifying contexts can ultimately yield similar effects, they represent two conceptually different constructs: whereas spacing aids memory, contextual diversity aids in creating a richer mental representation of the item.

These results are particularly important for cases in which exposure to the language itself is limited—as for example, in foreign language classrooms—and increasing the number of instances of a word is very costly. In addition, we show that the effect of diversity is not simply binary, but rather a gradient where more diversity leads to better outcomes. Prior studies had mostly focused on an all-or-none definition of diversity which did not give a clear picture of whether the effect increased passed an initial benefit. The current study shows that if contextual diversity is increased further, the benefits increase as well (at least from 8 repetitions in 8 texts to 8 repetitions in 1 text, as tested here).

We found no effects of language on comprehension, suggesting that the texts were equally understandable in both languages. Language only affected performance on the matching task but did not affect recall or recognition. Even if participants performed equally on lexical access tasks in both languages, they had a greater sense of familiarity with the correct meaning of the pseudowords in their native language. This allowed them to recognize better whether the pseudoword matched the image presented in their native language than their foreign one. Interestingly, our study provides a more nuanced picture of some of the differences between learning in a native or a foreign language. We see here that when the lexical items are matched between languages, they are equally difficult or easy to learn. This is in contrast with some previous literature that found that memory tends to be worse in a foreign language^23,46,47^, although these results are not very consistent^48^. This difference in results between memory for known vocabulary and new word learning suggests that either the effect is not very robust, or it does not extend to new vocabulary. Although not direct evidence, this is also somewhat in conflict with Pellicer-Sánchez’s^4^ findings that more exposures are necessary to reduce reading times in the foreign than the native language. Our results do suggest that perhaps their outcomes were partially caused by difficulties intrinsic to experience with the phonology or orthography of a language rather than to the language use itself. On the other hand, these effects could be influenced by the additional reading time in the foreign language context. Nevertheless, it should be kept in mind that this additional time within that condition correlated with worse performance. Also, even with the added reading time, we see that incorporating the item’s meaning is somewhat more difficult in the foreign language. These results also relate to and support those of Nassaji^10^ who found that the capacity to extract meaning from context relates to the knowledge of that language.

Importantly, there were no interactions between the main factors at study, showing that diversity had the same positive effect in both languages. Although against our initial hypothesis, this suggests that access to contextual information is enough to maintain the positive influence of diversity on word learning, despite the obvious difficulty of processing information in a non-native language. A prior study from the same authors found similar results with emotionality, where the effects of this variable were independent of language^49^. This supports the idea that the strategies for improving learning in the native language can apply to the foreign language, suggesting also that learning new vocabulary in one’s native and foreign languages engage similar mechanisms. Nevertheless, it should be noted that these participants had upper intermediate (B2) and above levels of English, thus allowing for the possibility that results might differ with low proficiency bilinguals.

These results have several implications. First, they qualify the value of contextual diversity across languages as well as generalize its importance, even overcoming difficulties in processing fluency. Second, they show that incidental vocabulary acquisition occurs similarly in a foreign and a native language. This also gives us a tool for improving this process by making strategic use of context-based spreading of information. Here, we show that it is not necessary to increase the number of exposures in order to improve learning, highlighting the importance of context and pointing to a perhaps overstated importance of frequency. It is worth noting that these results extend only to information recently learned and cannot speak to more long-term effects without further study. Nevertheless, this has important implications for education, where time and exposure are very limited and must be optimized. Future research should focus on possible mechanisms for these effects in order to understand their origin and extent. This paves the way for future studies focusing on how to affect context—or perhaps how novel words are presented in general—in order to improve incidental vocabulary learning.

## Supplementary information

Supplementary file1

## Data Availability

All data, scripts, and stimuli are available at https://osf.io/7ks4f/?view_only=5364dfadf99a41c283fa8b0c3a094453.
