# Supplementary material for: The effects of contextual diversity on incidental vocabulary learning in the native and a foreign language
Source: Sci Rep. 2020 Aug 18;10:13967. doi: 10.1038/s41598-020-70922-1 (PMC7435265; doi:10.1038/s41598-020-70922-1)
Supplement: Supplementary file 1 — Supplementary file1 [file 41598_2020_70922_MOESM1_ESM.pdf]

The effects of contextual diversity on incidental vocabulary learning in the native and a foreign language

Candice Frances[1*]

Clara D. Martin[1]

Jon Andoni Duñabeitia[2,3]

[1] BCBL, Basque Center on Brain, Language and Cognition; Donostia, Spain

[2] Centro de Ciencia Cognitiva – C3, Universidad Nebrija

[3] Department of Language and Culture, The Arctic University of Norway

*Contact information:

Candice Frances

c.frances@bcbl.eu

Basque Center on Cognition, Brain and Language (BCBL)

Paseo Mikeletegi 69, 2nd floor,

20009 Donostia - Spain

+34 943309300

Supplementary Table S1: **List of stimuli**

**Pseudo-word**	Category	English Word	Spanish Word	Distractor	Transposed Pseudoword	Transposed Distractor	Object Image	Category Competitor	Distractor Image	Unrelated Image
**nilan**	Beverage	water	agua	tesid	nisan	telid	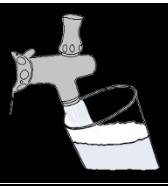	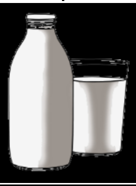	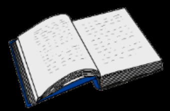	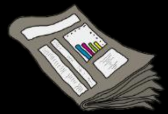
**etis**	Toy	ball	balón	omel	etes	omil	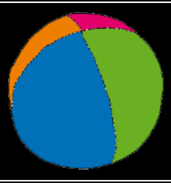	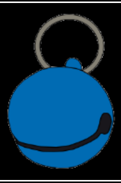	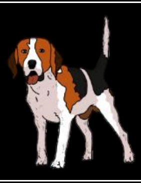	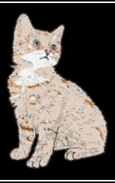
**esiba**	Dwelling	house	casa	iroma	esoba	irima	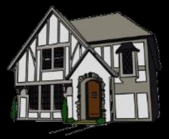	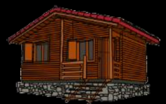	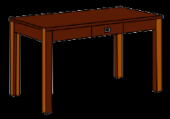	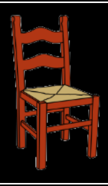
**asil**	Vehicle	car	coche	etis	atil	esis	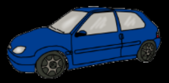	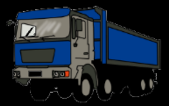	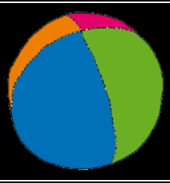	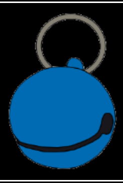
**tesid**	Reading material	book	libro	nilan	telid	nisan	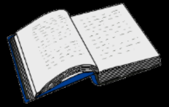	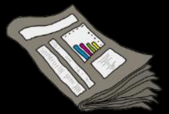	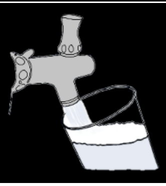	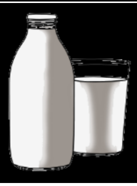

**otila**	Fruit	apple	manzana	esiba	esila	otiba	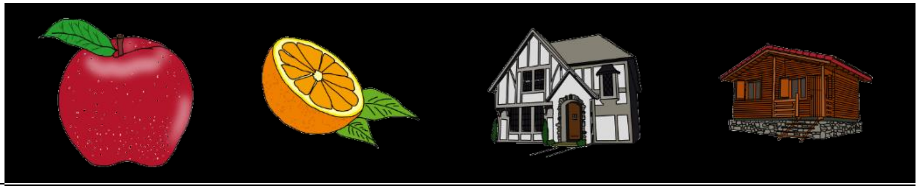
**iroma**	Furniture	table	mesa	otila	otola	irima	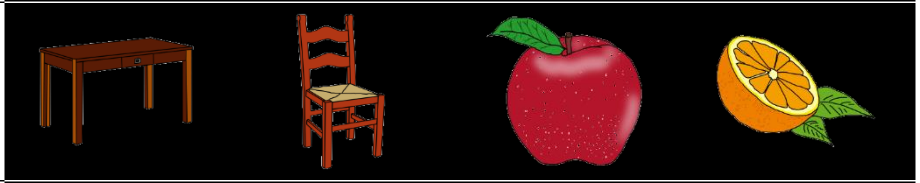
**omel**	Animal	dog	perro	asil	osel	amil	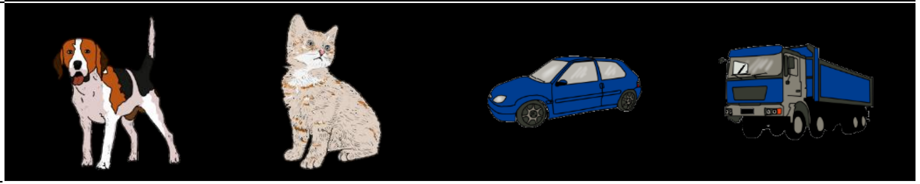

Supplementary Table S2**: List of key sentences**

Water

It was a long walk to see the _____ spring.

To avoid dehydration, they brought plenty of _____.

They stopped several times to drink _____ and have some snacks.

There was some _____, but almost no trace of the morning rain.

"Good thing we did not cancel for a few puddles of _____", she said.

The views were amazing, the _____ glowing in the sunlight as it hit the sand.

They walked past a small river with clear _____.

Past some trees, they reached the _____-fall.

Ball

"Come on! Pass me the _____!" they yelled.

She looked at the _____ and gave it a hard kick.

The _____ rolled past them further than she expected.

They all looked back and saw the _____ go right into the goal.

"Wow! You hit that _____ really hard!" they yelled surprised.

"Would you like to play with us?" he said pointing at the _____ .

"Sure. Pass me the _____ and get ready to catch!" she answered.

They kicked the _____ at her but when she tried to kick it back, she missed.

House

This was no ordinary _____: it was a home.

It was a brick _____ with a red roof.

She thought about moving to an apartment, but could not imagine selling the _____.

A _____ has to have strong foundations and a good roof.

Her _____ had that, plus a beautiful front with columns.

It seemed more like a mansion than a _____.

The _____'s fake marble floors and fancy lamps made it seem far more expensive than it was.

She had carefully chosen everything in that _____: from the furniture to the door frames.

Car

As soon as he sat, he smelled that new _____ smell.

He loved sitting behind the wheel of a new _____ .

He really enjoyed driving in general, but with this _____, it was really a pleasure.

He had already packed up the _____ for the road trip.

The idea was to test this _____ on a long road trip.

Even though it was a new _____, they checked the wheels, the engine, and the oil.

They "fed the _____"--as they called getting gas.

This made him think of his first _____--an old Ford.

Book

He loved reading _____s.

His friends preferred comics or graphic novels, but _____ were his favorite.

He would read the introduction or the first chapter of a _____ and get excited.

After finishing the _____, he would look up the author and the rest of his works.

The next step: finding similar _____ at the library.

He liked going through all the _____s on the shelf.

He wanted to be a novelist and write _____s for a living.

He had written some chapters for different _____s, but never finished any.

Apple

He was making caramelized _____s as he thought out loud.

"It is always bad to bite _____s in fairy tales like Snow white" he noticed.

"_____s are the forbidden fruit", he said.

As he got to the core of the _____, he found the seeds.

He would often be eating a sweet, juicy _____ and accidentally bit into a bitter seed.

“Better than a worm in my _____”, he thought.

He always removed seeds carefully when making _____ or orange juice.

"An _____ a day keeps the doctor away" he always said.

Table

These _____s and chairs are not easy to assemble he said.

The _____ had four legs and a strange board for the top.

It was more of a modern desk than an _____; so confusing.

"Can we just give up on this wooden kitchen _____?" he said.

"Just imagine this _____ with a beautiful tea set on top" she said.

He did not care about how the _____ would look in the living room.

He just wanted to be done with the _____ and chairs.

When they were finally done, it looked like a beautiful banquet _____.

Dog

He was out walking his _____.

As he walked, he thought about how _____s are a man's best friend.

He quickly became distracted when the _____ started barking.

He turned to see an _____ wagging his tail in their direction.

They approached as his _____ pulled on the leash to get closer to his new friend.

The two _____s smelled each other and started playing.

After his _____ seemed nice and tired, they walked away with his tongue hanging.

Sadly, this break lasted very briefly and his _____ was off chasing his tail in no time.

Supplementary Table S3: **Worked out example of stories**

1 text

"Come on! Pass me the etis!" they shouted. She looked at the etis and gave it a hard kick. The etis rolled past them further than she expected. They all looked back and saw the etis go right into the goal. "Wow! You hit that etis really hard!" they shouted surprised. "Would you like to play with us?" he said pointing at the etis. "Sure. Pass me the etis and get ready to catch!" she answered. They kicked the etis at her but when she tried to kick it back, she missed. She turned red and laughed as she joined them.

2 texts

"Come on! Pass me the etis!" they shouted. She looked at the etis and gave it a hard kick. The etis rolled past them further than she expected. They all looked back and saw the etis go right into the goal. She was quite pleased with herself as she sat down. She was talking to a boy she liked and he seemed pretty impressed, too. "I did not know you were good at sports," he said. "There is a lot you do not know about me," she replied. She liked flirting and acting mysterious, and sure enough, they all fell for her.

After lunch, everyone liked to hang out by the yard. She liked using this time to practice throwing. Sadly, today no one was playing. A group of boys that saw her the other day were coming over to talk. **"Wow! You hit that etis really hard!" they shouted surprised. "Would you like to play with us?" he said pointing at the etis. "Sure. Pass me the etis and get ready to catch!" she answered. They kicked the etis at her but when she tried to kick it back, she missed.** "Clearly, I'm much better at throwing than kicking," she said laughing.

4 texts

"Come on! Pass me the etis!" they shouted. She looked at the etis and gave it a hard kick. "Whoa! Grandma has some skill!" they shouted. She laughed as she moved past them. Although she was well in her 80's, she managed to stay surprisingly fit and healthy. She never worried about being thin or attractive, but she really enjoyed being active. She still goes for daily jogs and does yoga every day. Ever since she retired, she took up hiking and bicycling. She always told herself that being healthy should never be an effort and she sure lived by it.

She loved watching sports. There was always a sense of tension and expectation throughout**. The etis rolled past them further than she expected. They all looked back and saw the etis go right into the goal.** There was shouting and excitement from everyone. People cheering, hugging strangers, crying together with people they would never see again. She always felt there was something inexplicable that came over people. Something made them ignore social rules and just share their emotions. Surprisingly, after the game, the mask always comes back on and everyone returns to their normal lives and normal selves.

English class was always the best part of his day. Even though most of the activities were very silly, he liked that every day was different. Today, they were acting out a play. The play was about children in a school yard. **"Wow! You hit that etis really hard!" they shouted surprised. "Would you like to play with us?" he said pointing at the etis.** The dialogue was not the most interesting, but it was still pretty fun. Even though he was quite bad at English, he still enjoyed the activities. This class would always be his favorite.

It was her sixth month of physical rehabilitation. The first two months were terrible but things were getting better. Some days were good and others bad, but at least she could do more things. Rather than doing slow movements, she could now play some sports. She did it gently, but still could do it. Today, they asked her if she wanted to try something new. **"Sure. Pass me the etis and get ready to catch!" she answered.** **They kicked the etis at her but when she tried to kick it back, she missed.** Her body not responding meant it would not be a good day.

8 texts

"Come on! Pass me the etis!" they shouted. He ignored them and walked away. He hated the kids in the neighborhood. They were always outside where it was hot and they were so loud. They were all around his age but he had absolutely nothing in common with them. He liked video games and, unlike most kids his age, he really enjoyed studying. He could not tell anyone this, of course, because they would make fun of him. He would often spend full afternoons reading Wikipedia. Maybe he would do that today; it was too hot to be outside anyway.

"How do the neighbor's things keep getting in my yard?" she thought. **She looked at the etis and gave it a hard kick.** She then turned to the other neighbor's bike and rolled it to their yard. Cleaning up the front lawn had become a weekly ritual. She got tired of it always being messy with other people's things. "I guess that's the price of having neighbors," she thought. This time she was really annoyed, but not always. She had a busy week and did not feel like tidying up. "It needs to be done at some point, I guess," she mumbled.

This was the strangest film they had ever seen. There was a group of people playing and a woman commenting her thoughts. **The etis rolled past them further than she expected.** "What is this internal monologue?" he asked. "Honestly, I'm more confused about what she's wearing," she replied. "Is there even a plot here?" he replied back. "I actually think that this must have been recorded live," she said. "I'm scared for these people's mental health; they can't be completely sane if they made this," he said, laughing. They continued complaining, but secretly enjoyed how odd the film was.

Clearly, the soccer match was only an excuse to get together. They were all talking, eating and laughing. Basically, anything except watch the game. **They all looked back and saw the etis go right into the goal.** They made a weak attempt to celebrate and then went back to talking. They had not hung out like this since they were in high school. Now that they were about to finish college, it was going to happen even less. They were close, but sometimes life makes it hard to stay in touch. Deep down, they knew this would be the last time.

She never really liked children's birthday parties. It is especially bad when children are one or two years old. These parties end up full of adults just watching a baby. Everyone was being ridiculous. **"Wow! You hit that etis really hard!" they shouted surprised.** "That baby did not kick anything, this is ridiculous," she thought. Luckily, they always have wine at these events. She moved towards the bar to get a refill and act a bit social. All she could think about that night was how happy she was that she did not have to deal with kids.

It was the first day at a new school. Fifth grade is not easy for anyone, much less if you do not have friends. Play time was the most difficult. She was too scared to talk to anyone so she stood around trying to look busy. A boy was coming up to her, how embarrassing! **"Would you like to play with us?" he said pointing at the etis.** "Yes!" she said to him enthusiastically. Even though she was shy around boys, she had never been so relieved in her life. They played together and became the kind of friends that stay together forever.

Being the only American in the group was not easy. This meant that she had to constantly answer questions about her country. People asked her all kinds of questions. Her friends found everything about the US strange and they often had long discussions about it. Today, it was sports. "Can you show me how to play American sports?" one of them asked. **"Sure. Pass me the etis and get ready to catch!" she answered.** It was a nice day out and she did not mind doing a short demonstration. In the end, they spent the whole afternoon learning the little details of sports.

I think I'm going to be sick she said. Every single time they had physical education she felt ill. She hated the class and she was terrible at it. Plus, the teacher awful and mean. This time she was brave enough to go to class. She went, but still tried to hide in a corner, hoping no one would see her. Now they were all in a circle playing a strange game. **They kicked the etis at her but when she tried to kick it back, she missed.** She turned red and remembered why she never wanted to come to class.

Supplementary Table S4: **Non-parametric analyses of recall**

Given the lack of normality in the Recall data (both accuracy and ALINE distance), we carried out non-parametric tests.

First, for accuracy, we tested the effect of language for each level of diversity using a Mann-Whitney U test:

Diversity	Mann-Whitney U	*p*
8 Texts	958	0.929
4 Texts	880	0.364
2 Texts	946	0.812
1 Texts	968	1
Overall	897	0.541

This showed no effects of language for any particular level nor for the average of all levels.

Then, we carried out Friedman tests on diversity:

Language	Friedman Test Chi-Squared	df	*p*	Kendall's W
English	18.128	3	<.001	0.292
Spanish	17.133	3	<.001	0.318
Overall	34.713	3	<.001	0.306

We found significant effects of diversity for both languages and overall. We followed up the results of the overall test with post hoc Conover tests:

Conover's Post Hoc Comparisons - Diversity

		T-Stat	df	W_i	W_j	p_{holm}
8	4	2.600	261	255.000	224.000	0.030
	2	3.271	261	255.000	216.000	0.006
	1	5.872	261	255.000	185.000	< .001
4	2	0.671	261	224.000	216.000	0.503
	1	3.271	261	224.000	185.000	0.006
2	1	2.600	261	216.000	185.000	0.030

Note. Grouped by subject.

The same tests were carried out for ALINE distance. First, we tested the effect of language for each level of diversity using a Mann-Whitney U test:

Diversity	Mann-Whitney U	*p*
8 Texts	796	0.828
4 Texts	742	0.364
2 Texts	802	0.727
1 Texts	723	0.361
Overall	755	0.433

This showed no effects of language for any particular level nor for the average of all levels.

Then, we carried out Friedman tests on diversity:

Language	Friedman Test Chi-Squared	df	*p*	Kendall's W
English	24.321	3	<.001	0.579
Spanish	8.22	3	0.042	0.571
Overall	30.159	3	<.001	0.581

We found significant effects of diversity for both languages and overall. We followed up the results of the overall test with post hoc Conover tests:

Conover's Post Hoc Comparisons - Diversity

		T-Stat	df	W_i	W_j	p_{holm}
8	4	2.613	240	241.000	200.000	0.029
	2	1.816	240	241.000	212.500	0.141
	1	5.385	240	241.000	156.500	< .001
4	2	0.797	240	200.000	212.500	0.426
	1	2.772	240	200.000	156.500	0.024
2	1	3.569	240	212.500	156.500	0.002

Note. Grouped by subject.

Overall, these tests show a significant effect of diversity and no effect of language. Given that the results do not vary by diversity condition or by language, we can deduce that there is also no interaction between variables, as supported by the frequentist and Bayesian analyses.

Similarly for recognition accuracy, we ran the same analyses. First, we tested the effect of language for each level of diversity using a Mann-Whitney U test:

Diversity	Mann-Whitney U	*p*
8 Texts	825	0.120
4 Texts	956	0.913
2 Texts	982	0.890
1 Texts	1004	0.743
Overall	964	0.979

This showed no effects of language for any particular level nor for the average of all levels.

Then, we carried out Friedman tests on diversity:

Language	Friedman Test Chi-Squared	df	*p*	Kendall's W
English	11.153	3	0.011	0.253
Spanish	22.098	3	<.001	0.290
Overall	31.529	3	<.001	0.271

We found significant effects of diversity for both languages and overall. We followed up the results of the overall test with post hoc Conover tests:

Conover's Post Hoc Comparisons - Diversity

		T-Stat	df	W_i	W_j	p_{holm}
8	4	2.156	261	249	219.5	0.096
	2	1.023	261	249	235	0.517
	1	5.298	261	249	176.5	< .001
4	2	1.133	261	219.5	235	0.517
	1	3.142	261	219.5	176.5	0.007
2	1	4.275	261	235	176.5	< .001

Note. Grouped by subject.

As before, these tests show a significant effect of diversity and no effect of language. Given that the results do not vary by diversity condition or by language, we can deduce that there is also no interaction between variables, as supported by the frequentist and Bayesian analyses.

Supplementary Figure S5: **Screenshots of Learning**

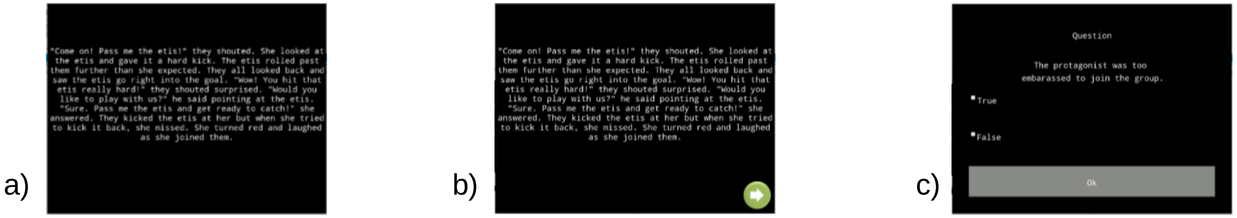

For the learning phase, (a) participants first had to read for 15 seconds before the “next” arrow appeared. (b) Once the arrow appeared, they were free to move on to the comprehension question at their own pace. (c) Once they pressed the arrow, they were presented with a true or false statement and were asked to select the correct answer.

Supplementary Figure S6: **Screenshots of Recall and Recognition Tasks**

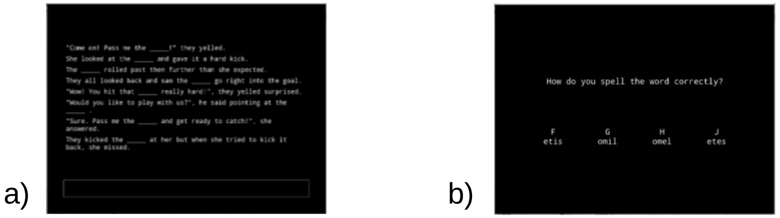

Figure 2: (a) Recall task: participants were presented with the eight key sentences and were asked to type in (once) the pseudoword. (b) Recognition task: once they pressed [enter], they were given the four possible answer options for which word could fill in the gap.

Supplementary Figure S7: **Screenshots of Matching Task**

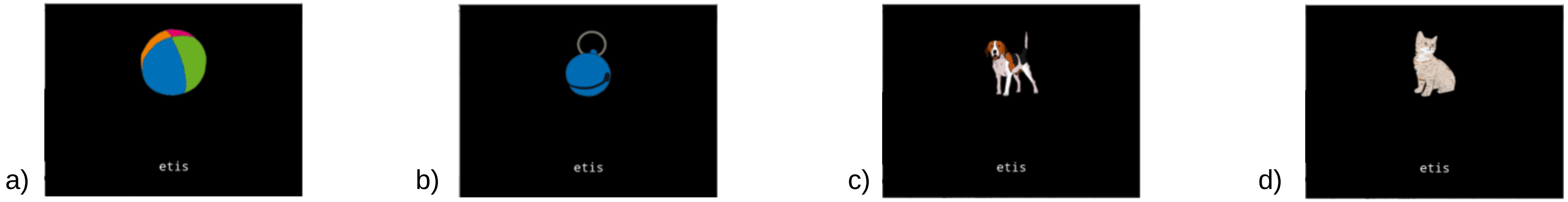

The matching task presented either (a) the correct match, (b) a category competitor, (c) the image of another keyword, or (d) an unrelated word (which was the category competitor of the other keyword).
